# Developmental Aspects of Glucose and Calcium Availability on the Persistence of Memory Function Over the Lifespan

**DOI:** 10.3389/fnagi.2019.00253

**Published:** 2019-09-11

**Authors:** Matthew R. Holahan, Niko Tzakis, Fernando A. Oliveira

**Affiliations:** ^1^Department of Neuroscience, Carleton University, Ottawa, ON, Canada; ^2^Laboratory of Cellular and Molecular Neurobiology (LaNeC), Center for Mathematics, Computing and Cognition, Federal University of ABC (UFABC), São Bernardo do Campo, Brazil

**Keywords:** childhood, energetic metabolism, calcium, memory, neurodegeneration, early life experience, aging, glucose

## Abstract

An important aspect concerning the underlying nature of memory function is an understanding of how memories are acquired and lost. The stability, and ultimate demise, of memory over the lifespan of an organism remains a critical topic in determining the neurobiological mechanisms that mediate memory representations. This has important implications for the elucidation and treatment of neurodegenerative diseases such as Alzheimer’s disease (AD). One important question in the context of preserving functional plasticity over the lifespan is the determination of the neurobiological structural and functional changes that contribute to the formation of memory during the juvenile time frame that might provide protection against later memory dysfunction by promoting the establishment of redundant neural pathways. The main question being, if memory formation during the juvenile period does strengthen and preserve memory stability over the lifespan, what are the neurobiological structural or functional substrates that mediate this effect? One neural attribute whose function may be altered with early life experience and provide a mechanism to preserve memory through the lifespan is glucose transport-linked calcium (Ca^2+^) buffering. Because peak increases in glucose utilization overlap with a timeframe during which spatial training can enhance later memory processing, it might be the case that learning-associated changes in glucose utilization would provide an important neural functional change to preserve memory function throughout the lifespan. The glucose transporters are proteins that are reduced in AD pathology and there is evidence that glucose reductions can impair Ca^2+^ buffering. In the absence of an appropriate supply of ATP, provided via glucose transport and glycolysis, Ca^2+^ levels can rise leading to neural vulnerability with ensuing pathological outcomes. In this review, we explore the hypothesis that enhancing glucose utilization with spatial training during the preadolescent period will provide a functional enhancement that regulates glucose-dependent Ca^2+^ signaling during aging or neurodegeneration and provide essential neural resources to preserve functional plasticity and memory function.

## Overview

During development, the central nervous system exhibits an extremely growth- permissive intra- and extra-cellular milieu (Benowitz and Routtenberg, 1997; Coleman, 2005; Muramatsu et al., 2009) whereby formation of new synapses and neural circuits are remodeled as the brain strengthens active connections and eliminates others (Yiu and He, 2006). Brain-wide or localized remodeling often occurs during critical or sensitive periods (Fenoglio et al., 2006) when a regular occurrence of pruning and outgrowth lead to the adult configuration (Kantor and Kolodkin, 2003). The formation of new synapses and the elimination of others leads to highly efficient neural networks that allow for the emergence of complex integrative functions. The synergistic action of genetic factors and responses to external stimuli (Sur and Rubenstein, 2005) leave the brain in a highly malleable condition during these times (Anderson et al., 2011). If the brain is adversely affected by environmental insults during sensitive developmental periods, development may be compromised with long-term detrimental effects throughout the lifespan (Shonkoff et al., 2009). However, beneficial environmental events can have long-lasting adaptive outcomes that enhance neural functioning (Kempermann, 2019).

### Functional Plasticity

Plasticity describes how the mammalian central nervous system shows adaptive changes in organization and function with experience (Citri and Malenka, 2008; Bernardinelli et al., 2014; Maren, 2015). Given the ability of neural synapses and networks to respond to a variety of experiences, it is hypothesized that plasticity during development may represent a fundamental property for adaptive plasticity throughout the lifespan (Caroni et al., 2014). Early neurodevelopmental plasticity can be hypothesized to be beneficial and associated with successful adaptation to a wide range of environmental experiences during the lifespan.

Functional plasticity can be used to describe synaptic modifications, such as neurotransmitter release or receptor changes, that influence the likelihood of signal propagation (Kerchner and Nicoll, 2008; Granger and Nicoll, 2014). Synaptic phenomena such as long-term potentiation (LTP) and long-term depression (LTD) would be considered the epitome of functional plasticity. Functional plasticity is associated with changes in intracellular calcium (Ca^2+^) concentrations mediated by voltage-gated Ca^2+^ channels (VGCCs; Verkhratsky and Toescu, 2003; Verkhratsky and Parpura, 2014) or synaptic glutamate binding to *N*-methyl-D-aspartate receptors (NMDAR) (Park et al., 2014). Rising levels of intracellular Ca^2+^ initiate second messenger cascades (MacDonald et al., 2006) which can result in the induction of LTP. If repeated rises in intracellular Ca^2+^ occur as would happen during development, high-frequency stimulation or learning, functional plasticity may lead to long-term permanent changes in synaptic function and stability of neural networks.

### Glucose Metabolism

Glucose is a carbohydrate that fuels neural activity to permit functional and structural changes in neurons associated with plasticity and memory processes. Glucose metabolism serves a variety of critical roles required for the central nervous system to show functional plasticity (Magistretti, 2006; Pearson-Leary and McNay, 2016) as well as limit the pathological processes that might ensue from high levels of intracellular Ca^2+^ (Dienel, 2019). Because glucose hypometabolism is a contributing factor during pathological neural responses like those that culminate in neurodegenerative diseases such as Alzheimer’s disease (AD; Simpson et al., 1994; Duran-Aniotz and Hetz, 2016; Daulatzai, 2017; An et al., 2018), enhancing glucose utilization during critical stages of development may serve to protect against neural decline. Because Ca^2+^ is a pivotal ion for learning and memory and is crucial for a variety of neuronal signaling pathways (Berridge et al., 2000; Art, 2012; Carafoli and Krebs, 2016), energy-dependent regulatory mechanisms are fine-tuned to regulate tight control over levels of intracellular Ca^2+^. In the absence of appropriate energy supply, Ca^2+^ levels can rise with ensuing pathological outcomes (Mattson, 2007).

## Precis

In this review, we summarize a sensitive developmental period for the emergence of spatial/allocentric processing in both humans and rodents. This developmental period is reflected in remodeling of hippocampal networks that may provide the neural substrate for these behavioral changes (Comba et al., 2015). This period appears to be significant as changes that occur during this timeframe may well influence information processing capabilities throughout the lifespan. As this period of functional plasticity would require changes in intracellular Ca^2+^ levels, the ability to regulate intracellular Ca^2+^ is critical. Our central tenet is that glucose metabolism and utilization provide the necessary energy for intracellular Ca^2+^ regulation. While immediate changes in glucose-mediated Ca^2+^ regulation might not have an acute impact on functional plasticity during this time, the long-term outcome may manifest during aging, leaving these hippocampal circuits vulnerable to neurodegenerative processes. Therefore, glucose metabolism during this postnatal developmental time frame establishes appropriate neural substrates that are essential to preserving functional plasticity throughout the lifespan and any dysregulation of glucose metabolism could result in pathological processes and memory decline throughout the lifespan.

## Developmental Definitions

To determine potential sensitive developmental periods for central nervous system functional plasticity, it is important to outline various milestones that might reflect changes in neural function (see Table 1). In general, three different stages of development are thought to be conserved across a number of mammalian species: (i) childhood (juvenile; preadolescence; in humans, approximately 0–12 years; in rodents, average age to attain puberty is 42 days; Dutta and Sengupta, 2016) is a time when animals develop neural circuits to subserve essential sensory and motor functions to properly interact with an ever-changing environment; (ii) adolescence (in humans, approximately 12–21 years; in rodents, sexual maturity is attained on average at 10 weeks; Dutta and Sengupta, 2016) is the start of puberty when independence and social skills are developed and fine-tuned; and (iii) adulthood (in humans, over 21 years) when physical growth is nearly complete and intellectual maturity has been achieved (Piekarski et al., 2017).

**TABLE 1:** Comparison of the main developmental milestones in humans, rats and mice with approximate ages based on studies cited.

**Species**	**Childhood/preadolescence/prepuberty/juvenile^∗^**	**Adolescence/sexual maturity**	**Adulthood/sexual maturity to reproductive senescence^∗∗∗^**
**Human**		Kilborn et al., 2002	Sengupta, 2013
Males	0 to 108 ± 2.0 months^∗∗^	Range from 108 to 168 months to 204 (17 years) to 240 (20 years) months	
Females	0 to 108 ± 1.8 to 144 ± 1.2 months	96–156 months 180 (15 years) to 240 (20 years) months	Menopause average of 51 years
**Rat**		Sengupta, 2013	Sengupta, 2013
Males	0–36.5 ± 1.1 days	35–48 days to 210 days	
Females	0–37.5 ± 2.7 days	32–39 days to 210 days	Reproductive senescence from 15 to 20 months
**Mouse**		Kilborn et al., 2002	Dutta and Sengupta, 2016
Males	0–36.5 ± 1.1 days	35 days to 150 days	
Females	0 to 29.12 ± 2.4 to 41 ± 2 days	31 days to 150 days	Reproductive functions cease around 15 months

*In the Kilborn et al. (2002) publication, differences in the duration of bone growth and its relationship to age at sexual maturity and lifespan were main indices used for comparison. Sengupta (2013) and Dutta and Sengupta (2016) concluded that for accurate comparisons between humans and rodents, each phase of life needs to be taken into account rather than simply the entire lifespan. ^∗^Data from citations in text under section “Developmental Definitions” unless otherwise noted. ^∗∗^Data are expressed as mean ± standard deviation. ^∗∗∗^Reproductive senescence used as an indicator of the upper limit of adulthood prior to onset of senescence.*

The childhood period (0–12) is pivotal in humans (and other animals) and is characterized by rapid, age-related changes in size and abilities when several neural networks are refined (growth and pruned) to facilitate and reflect enhanced experience-dependent functional plasticity (van Dyck and Morrow, 2017). During the childhood period, the majority of neural rearrangements that take place occur at the local, synaptic level, as opposed to the global or network level (Freitas et al., 2013), to optimize immediate sensory and motor adaptations to the natal environment. During this period of development, children learn complex skills that will form the foundation for adapting to complex social and cultural experiences during the lifespan (Shonkoff and Phillips, 2000; Bock and Sellen, 2002; Konner, 2010). Most humans learn to speak, read, write and perform more complex cognitive skills which are thought to rely on local, synaptic modifications in the neocortex. This high degree of functional plasticity in childhood underlies an enhanced learning capacity and potentially sets up the foundations for network plasticity throughout the lifespan.

The onset of adolescence (approximately 12 years of age in humans) represents a transition from a high preponderance of local synaptic changes during childhood to a balance between local synaptic changes and global network changes in brain function (Freitas et al., 2013). Typically, the onset of the adolescent phase and the end of the childhood period occurs with puberty (Juraska and Willing, 2017). In humans, data have shown that at the mean age of 9.5 ± 1.8 (standard deviation; SD) years, girls start showing the first signs of puberty with the first menarche happening around 12.5 ± 1.2 (SD) years (Herman-Giddens et al., 1997; Piekarski et al., 2017). For boys, the first sexual signs indicating the onset of puberty (and, adolescence) start with a mean age of 9.7 ± 2.0 (SD) years of age (Herman-Giddens et al., 2012; Piekarski et al., 2017). In female mice, the first sexual signs of puberty start at 29.12 ± 2.4 (SD) days and the first estrus (menarche equivalent) at 41 ± 2 (SD) days (Piekarski et al., 2017) while male mice start with the first signs of puberty at the age of 36.5 ± 1.1 (SD) (Deboer and Li, 2011). Female rats display sexual signs for puberty starting at 37.5 ± 2.7 (SD) days with first estrus at 37.8 ± 2.7 (SD) days. In male rats, the first signs of puberty occur at 36.5 ± 1.1 (SD) days (Korenbrot et al., 1977; Clark and Price, 1981).

## Cognitive Development

Piaget (1936) is credited as one of the first psychologists to make systematic observations of child cognitive development and formalize various stages of cognitive abilities. He completed a series of simple yet original investigations to categorize different stages of cognitive abilities in children from birth to 11 years old (Inhelder and Piaget, 1958). While there are valid criticisms of his methods and conclusions (McLeod, 2018), his four stages of cognitive development do provide a worthwhile starting point for the exploration of developmental abilities. During the preoperational stage, 4–6-year-old display egocentric tendencies whereby they are only capable of considering the world from their own perspective. In a test of visual-spatial awareness (mountains study), Piaget placed children in front of a model mountain range and asked them to pick from a selection of pictures that Piaget would see. Children that were 7 and younger chose the viewpoint that they saw and therefore lacked the ability to appreciate a viewpoint different from their own (egocentric). During the concrete operational stage (7–12), children became less egocentric and seemed to appreciate viewpoints other than their own now being able to engage in cognitive perspective-taking (allocentric). When comparing Piaget’s stages to childhood and adolescent periods, the preoperational and concrete operational stages define the childhood period.

### Development of Allocentric Abilities in Humans

During development, humans make use of both egocentric and allocentric strategies when processing information. Egocentric strategies are frameworks of the environment that move with the self, while allocentric strategies are frameworks of the environment that remain fixed and do not change as a function of personal movement (Burgess, 2006; Evans et al., 2016; Colombo et al., 2017; Boutet et al., 2018). In Piaget’s perspective, children 7 and younger rely on egocentric information while children from 7 to 12 begin to use allocentric information (Inhelder and Piaget, 1958). Forming a cognitive map of the environment, as is accomplished in allocentric processing, requires the integration of landmarks in the environment and depends on hippocampal function (O’Keefe and Nadel, 1978; Bohbot et al., 2004; Nadel and Hardt, 2004; Chersi and Burgess, 2015). Imaging studies have revealed that allocentric information processing is associated with elevated hippocampal activity (Bohbot et al., 2004; Parslow et al., 2005; Zaehle et al., 2007) and patients with hippocampal damage show impaired performance on tasks that require allocentric processing (Holdstock et al., 2000; Bohbot et al., 2004; Feigenbaum and Morris, 2004).

**FIGURE 1:**
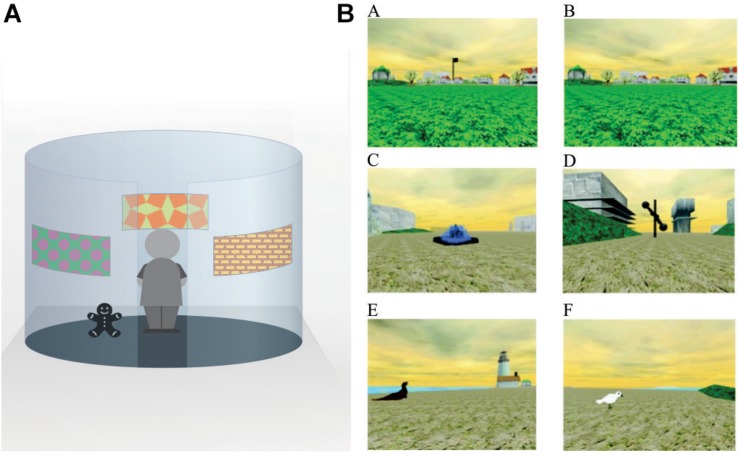
Examples of spatial tasks used to assess allocentric processing in humans. **(A)** An example of a circular arena using indirect cues to locate a goal. In the indirect test, the target object (bear in figure) remains at a fixed location in relation to the indirect cues (images on the walls). The indirect cues provide information as to where the target object is located but no one cue indicates the precise location of the object. **(B)** Screen shots from the Memory Island Navigation Task (open access from https://openi.nlm.nih.gov/detailedresult?img=PMC3906800_abn_122_4_1189_fig1a&req=4). For this task, subjects complete both visible **(A)** and hidden trials **(B)**. In the visible condition, target items **(C–F)** are marked by large flags, which can be seen from far away. In the hidden condition, no flag markers are present and subjects are required to locate the same target object **(D)** on each trial (the location is identical to the visible trial). The hidden trials test for allocentric/spatial learning.

In Piaget’s original expositions, the ability to integrate and coordinate relational aspects of place (allocentric learning) emerged in 9 and 10-year-old (Inhelder and Piaget, 1958). One study assessed spatial memory and orientation in children (5, 7 and 10 year olds) with the Kiel Locomotor Maze allowing the determination as to whether children used a cue strategy (goal localization with a local cue close to the goal) or a place strategy, that relied on the ability to integrate distal sensory cues for orientation (Lehnung et al., 1998). The authors reported that during testing, the 5-year-old relied only on a cue strategy, orientating to local cues. The 10-year-old, on the other hand, used distal cues for orientation relying on a place strategy. Interestingly, half of the 7-year-old used a non-spatial strategy while the other half used a place strategy (Lehnung et al., 1998). In another study, the emergence of cue and place learning and retention were examined in six age groups (3, 4, 5, 7, 10 and 12 years) using three different tasks (radial arm maze, Morris water maze, and open-field search-task) (Leplow et al., 2003). Overall results revealed that place learning was fully developed by the age of 10 whereas participants up to the age of 7 relied on cue strategies. In a similar study, 7 and 10-year-old children were tested on an object location memory task (Bullens et al., 2010). In this study, two conditions were assessed: the direct cue condition associated the target object with a specific landmark cue to test non-spatial learning; the indirect cue condition associated the target object with distal landmarks surrounding the arena to measure place learning (see Figure 1A). Results revealed the 7-year-old children performed significantly worse than 10-year-old in the place learning task whereas both age groups performed similarly in the non-spatial test condition (Bullens et al., 2010). The Memory Island virtual maze task is a human spatial memory assessment tool modeled after the Morris water maze (see Figure 1B). Using this task, Piper et al. (2010) noted a considerable improvement in spatial memory from ages 7 to 10. Because the distance traveled to reach the targets was not affected by age, the authors suggested that the differences in spatial memory were not due to age-associated differences in task performance (Piper et al., 2010).

While none of these studies reported sex differences, one study found that 9-year-old boys were significantly faster to acquire and retain a virtual hidden platform water maze task then 9-year-old girls (Newhouse et al., 2007). The authors reported a lack of sex differences in navigating to the virtual visible platform ruling out any performance factors. An interesting conclusion from this study is that these sex differences in spatial processing are present prior to puberty and are therefore not due to changes in sex hormones that occur with the onset of puberty (Newhouse et al., 2007). In a similar vein, a sex difference was noted on the virtual Memory Island task such that males outperformed females at all age groups (6–11, 12–17, and 18–39; the 40–67-year-old sex effect was not significant) (Piper et al., 2011). Once again, sex hormones were ruled out as the sole factor because differences in spatial processing were found in both pre- and post-pubescent groups. Consistent with the above-cited studies, this study also revealed a significant improvement in spatial processing in the 12–17 group compared to the 6–11-year-old group. A detailed presentation of the ages showed that this improvement in spatial memory occurred around 11–12 years of age (compared to 10 and under; for details see Figure 1 in the paper) (Piper et al., 2011).

### Development of Allocentric (Spatial) Abilities in Rodents

**FIGURE 2:**
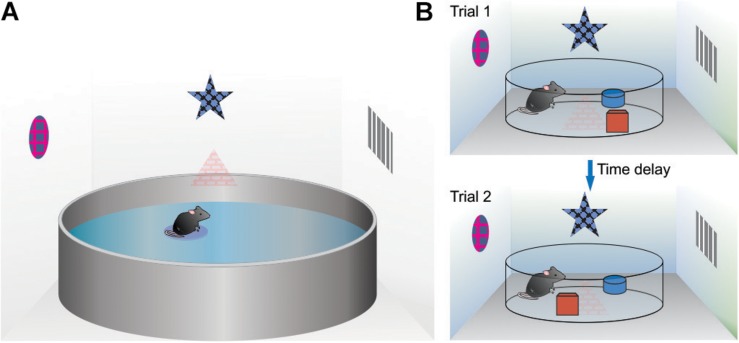
Examples of spatial tasks used to asses allocentric processing in rodents. **(A)** The water maze task. The basic procedure consists of placing a rodent (mouse or rat) into a large circular pool filled with opaque water. In the spatial version, the animal is required to swim to a platform that is slightly submerged under the water making it hidden from view. The rodent locates the platform (and remembers its location) by using various cues placed around the room (indirect cues). **(B)** For the object in a novel location spatial task, rats are individually placed into an arena and allowed to explore. Two objects are placed in different quadrants and remain in the same location for the training phase. At some time delay after training, rats are tested whereby one of the objects is moved to a different quadrant of the arena. This procedure takes advantage of a rats spontaneous tendency to explore objects that have changed location within an otherwise stable environment.

Converging lines of evidence from a number of behavioral studies using rats point to the postnatal period (PND) from 19 to 21 days as a sensitive developmental timeframe for the emergence of spatial behavioral function. Using the hidden- and visible-platform versions of the water maze task, PND19 rats showed improved performance on the visible platform task (compared to PND17) but lacked spatial abilities as evidence from a lack of improvement across training trials and lack of 24-h retention on the probe test (Rudy et al., 1987). At PND21, performance on the hidden-platform task (see Figure 2A for task overview) was noted during acquisition compared to the PND19 rats but also, intact spatial memory retention as evidenced by the increased amount of time spent in the target quadrant during the 24-h probe test (Rudy et al., 1987). In tests of contextual fear conditioning, that rely on spatial information processing, on the conditioning day, PND18 rats showed as much freezing to the auditory cue as PND23 rats but PND18 rats displayed much less freezing to the training context than PND23 rats (Rudy, 1993). A follow-up study showed minimal freezing in the conditioned context at a 24-h retention interval in the PND18 rats compared to significant freezing in the PND23 rats (Rudy and Morledge, 1994; Pugh and Rudy, 1996). These results indicate that spatial-contextual acquisition and retention emerge between PND18 and PND23 (Raineki et al., 2010) and as early as PND17 (Nair and Gonzalez-Lima, 1999).

While initial behavioral studies using spatial alternation suggested spatial function emerged around PND28 (Douglas et al., 1973), a number of subsequent studies measuring spatial navigation on the water maze (Tonkiss et al., 1992; Kraemer and Randall, 1995; Brown and Whishaw, 2000; Akers and Hamilton, 2007; Akers et al., 2011), working memory (Green and Stanton, 1989), spatial alternation (Dumas, 2004; Blair et al., 2013) and the use of contextual representations in the development of fear responses (Jablonski et al., 2012) point to the emergence of spatial function being around PND20. Optimal performance on a delayed alternation task has been shown to emerge between PND19 and PND27 but in pups with PND10 hippocampal lesions, this behavior fails to fully develop (Freeman and Stanton, 1991) suggesting that hippocampal integrity is important for the normal development of spatial learning and other brain structures do not compensate when the hippocampus is damaged during postnatal development (Altemus and Almli, 1997). Likewise, PND22–PND24 rats performed a spatial alternation task significantly better than PND17–PND19 rats indicating optimal hippocampal function by PND22 (Blair et al., 2013).

On both place and cued-place water maze tasks, PND19 and PND20 rats showed good learning and probe trial responses whereas PND18 rats did not (Brown and Whishaw, 2000). As well, with only distal cues present, PND20 and PND24 but not younger rats learned the location of a hidden platform (Akers and Hamilton, 2007). Using male and female Long Evans rats, spatial memory function was assessed in a water maze task from PND16 to PND26. From PND16 to PND19, the stable performance was observed with moderate improvements in locating the hidden platform. From PND19 to PND21, there was a dramatic improvement in locating the hidden platform to levels seen in adult Long Evans rats (LER; Keeley et al., 2010; Wartman et al., 2012a). In the Keeley et al. (2010) report, there were no male-female sex differences found in the acquisition or retention of the spatial information.

Behavioral results from Comba et al. (2015) revealed that PND20 rats given 1 day of hidden-platform water maze training had significantly lower latencies than the groups trained on PND16 or PND18 suggesting the emergence of spatial function by PND20. However, the PND20 group also showed significantly faster swim speeds indicating that better water maze performance in the PND20 group could be due to physicality rather than spatial/cognitive function. To examine whether the improvements seen at PND20 were due to cognitive/spatial or motor development, an experiment was run on a dry maze (see Figure 2B for overview). This task is less physically demanding relying on exploration rather than swimming (Schapiro et al., 1970; Altman and Sudarshan, 1975), and thus was hypothesized to rely more on cognitive functions. For the spatial dry maze task, the rats performed significantly better than chance at PND19 and PND20 but younger rats (PND16, PND17, PND18) showed chance performance (Comba et al., 2015). Because the dry maze task takes advantage of a rat’s ability to detect changes in the spatial relationship between the two objects but is physically less demanding than the water maze task (Mumby et al., 2002; Gaskin et al., 2009), a preliminary conclusion is that spatial-cognitive behavioral function emerges around PND19/PND20, consistent with numerous other studies.

Studies in other rodent species have revealed a similar developmental time course for the emergence of allocentric-spatial processing abilities. In one study (Chapillon and Roullet, 1996), three learning procedures were used (place learning; cue learning; cue + place learning) with PND 22 mice. Results revealed that PND22 mice showed the same capabilities as adults and showed the ability to integrate distal cues by showing a strong spatial bias during a probe test. Using the hidden platform water maze task, short- and long-term memory retention was assessed in mice that ranged in age from PND15 to PND150 at the beginning of training (Guskjolen et al., 2016). All age groups showed spatial memory when tested 1 day after training, but only PND20 and older mice showed spatial recall one month later. Careful assessment of the memory deficit in the younger mice revealed that it was not due to weaker encoding and was likely due to a retrieval, rather than storage, deficit (Guskjolen et al., 2016). In a study examining the spatial processing capacities of meadow voles, there were significant differences in acquisition rate of the spatial task between PND20 and PND10 and PND15 voles (Galea et al., 1994). This suggests the emergence of allocentric-spatial processing abilities at PND20 in the meadow vole.

### Human-Rat Comparison of Developmental Emergence of Spatial-Allocentric Ability

**FIGURE 3:**
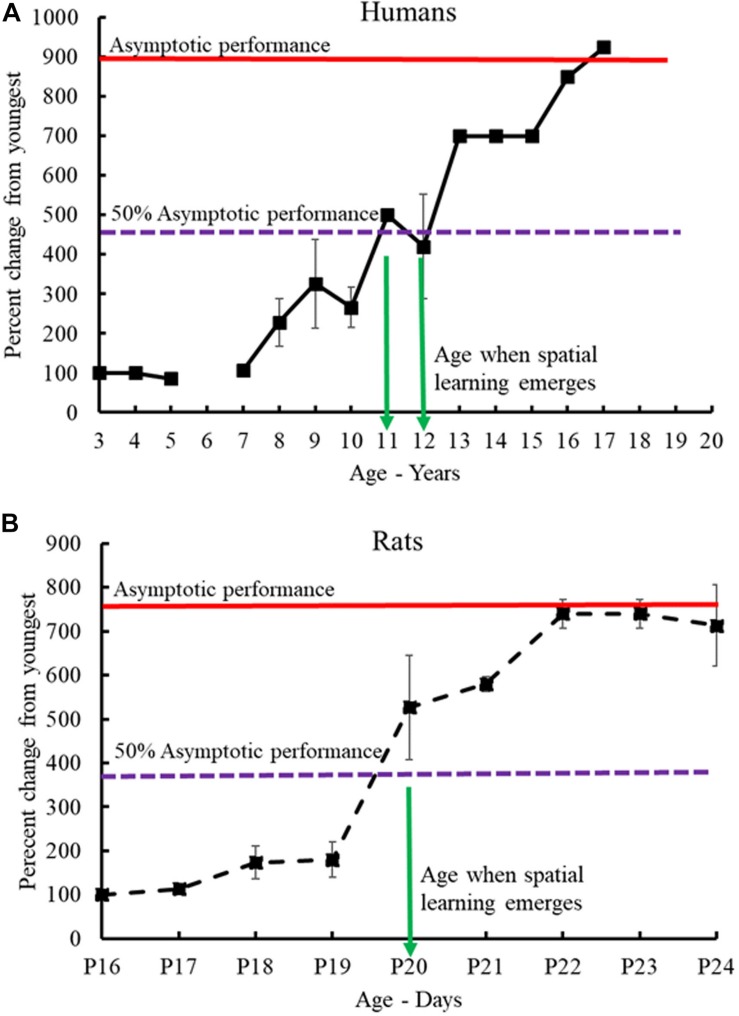
Human-rat comparison of developmental emergence of spatial-allocentric ability. Data from the human **(A)** and rat **(B)** studies (Table 2) were used to illustrate the developmental time when spatial memory function emerged in both species. Data from each paper from the youngest age group included in the study were used as the baseline value. These values were converted to 100%. From that, the change from baseline for each of the ages as reported in the studies up to the oldest age groups was determined. The red line indicates asymptotic performance as reported in the studies and from this value, the half maximal value (50% of asymptotic performance – dashed purple line) was calculated to determine the age at which allocentric-spatial learning emerges. Allocentric processing appears to emerge in 11/12-year-old humans **(A)** and spatial processing appears to emerge in rats **(B)** at PND20/PND21 (green arrows).

Compiling the data from the human and rat studies (mice and vole studies excluded) as cited above (see Table 2 for summary and Table 3 for analysis), spatial-allocentric learning appears to emerge in humans around 11/12 years of age and in rats, around PND20/PND21 (Figure 3). In a separate line of investigation, we took the data reported from the human and rat studies included above and developed a “memory function curve.” Data from each paper from the youngest age group included in the study were used as the baseline value. These values were converted to 100%. From that, we calculated the change from baseline for each of the ages as reported in the studies up to the oldest age groups. This allowed us to present a graphic of the change from the youngest group over time and generate an asymptotic performance level (Figure 3, red line). From the asymptotic performance level, we calculated the half maximal value (50% of asymptotic performance – Figure 3, dashed purple line) to determine the age of allocentric-spatial emergence. As can be seen in Figure 3, the age corresponds to the data cited in the studies whereby, allocentric processing appears to emerge in 11/12-year-old humans and spatial processing appears to emerge in rats at PND20/PND21 (Figure 3, green arrows).

**TABLE 2:** Studies used to generate data for Figure 3.

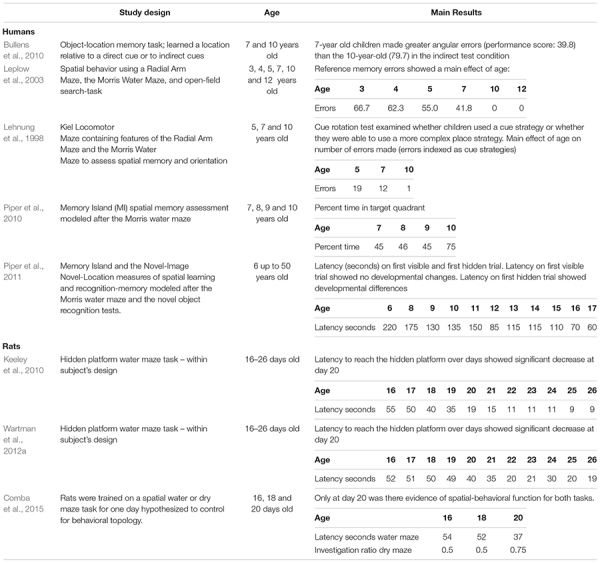

*The aim of collecting these studies was to allow comparisons of spatial/allocentric processing across early developmental time periods that corresponded to the developmental time windows described in Table 1.*

**TABLE 3:** Data from the human and rat studies (extrapolated from studies listed in Table 2) were used to illustrate the developmental time when spatial memory function emerged in both species.

**Humans**	**Age – Years**														
	
	**3**	**4**	**5**	**6**	**7**	**8**	**9**	**10**	**11**	**12**	**13**	**14**	**15**	**16**	**17**
	100	100	42.85		100			228.57		228.57					
			100		134			200							
			100		126			180							
					100			270.96							
			100		100										
					100	106.66	102.22	166.66							
					100					182.03					
					100	350	550	550	500	850	700	700	700	850	925
**Average**	**100**	**100**	**85.71**		**107.5**	**228.33**	**326.11**	**266.03**	**500**	**420.20**	**700**	**700**	**700**	**850**	**925**
***SEM***			**10.10**		**4.96**	**60.83**	**111.94**	**50.91**		**131.85**					

**Rats**	**Age – Postnatal day**														
	
	**12**	**13**	**14**	**15**	**16**	**17**	**18**	**19**	**20**	**21**		**22**	**23**		**24**

						100	100	100	700						900
					100	120	200	220	580	600		780	780		620
					100	120	220	220	300	560		700	700		620
**Average**					**100**	**113.33**	**173.33**	**180**	**526.66**	**580**		**740**	**740**		**713.33**
***SEM***					**0**	**6.66**	**37.11**	**40**	**118.50**	**16.32**		**32.65**	**32.65**		**93.33**

*Data from each paper from the youngest age group included in the study were used as the baseline value. These values were converted to 100%. From that, the change from baseline for each of the ages as reported in the studies up to the oldest age groups was determined.*

## Neural Substrates That Support the Emergence of Allocentric-Spatial Function

One brain region that shows connectivity-based changes during sensitive, postnatal developmental periods and plays a critical role in allocentric-spatial processing is the hippocampus. Based on the behavioral data above, the hippocampus would be predicted to show anatomical and functional changes that overlap with the PND20/PND21 period in rats and, by extension, the 11/12-year-old period in humans. There are numerous studies on the rat that provide support for this contention (see references in the following sections).

### Axonal Remodeling

Neurogenesis in the dentate granule cell layer shows peak density labeling as early as PND7/PND8 (Schlessinger et al., 1975) and more consistently at PND13/PND15 (Altman and Das, 1965; Cahill et al., 2017) and between PND19 and 25, the number of granule cells increases dramatically (Bayer, 1980). More recent results show that neurogenesis in the dentate gyrus shows adult-like levels by PND21 (Ciric et al., 2019). In conjunction with this, the mossy fibers, forming the connections between the granule cells of the dentate gyrus and the pyramidal cells of the CA3 subfield, show a late, postnatal period of remodeling (Gaarskjaer, 1985, 1986). Using the Timm’s stain to analyze the mossy fiber projection in LER rats, a mossy fiber projection to the *stratum oriens* was seen to develop between PND18 and PND21. By PND21/PND24, the projection to the *stratum oriens* resembled that seen in the adult (Amaral and Dent, 1981; Holahan et al., 2006, 2007). The pattern of synaptophysin staining, indicative of axonal terminals, in the CA3 hippocampal region, showed a similar developmental emergence and pattern (Comba et al., 2015) suggesting the hippocampus undergoes a period of remodeling that spans PND19–PND21.

### Electrophysiological Plasticity

A number of electrophysiological studies are consistent with the hypothesis that the emergence of hippocampal-based spatial function occurs around the PND18–PND21 timeframe. Place cells with specific spatial firing patterns have been recorded as early as PND17 with place cell patterns conveying optimal spatial information showing continued development up to PND35 (Langston et al., 2010; Ainge and Langston, 2012). Likewise, place cell firing is present from PND16 to PND26 but continues to improve throughout development with stable place cell recordings (i.e., similar to adults) being made at PND28 (Wills et al., 2010). Theta-modulated firing is present at PND16 in the hippocampus CA1 region and these responses reach adult levels by PND22 (Langston et al., 2010; Wills et al., 2010). After PND21, both the magnitude and threshold for post-synaptic induction of LTP are reduced with a corresponding increase in the threshold for presynaptic induction (Dumas, 2012; Blair et al., 2013).

### Vulnerability and Benefits

Additional support for the idea that PND19–PND21 is a sensitive developmental period for hippocampal-based functioning comes from work showing (1) administration of an NMDA-receptor antagonist from PND17 to PND20 impairs the development of the mossy fiber projection to *stratum oriens* in Long Evans rats (Holahan et al., 2007) (2) training on spatial water or dry maze tasks during this period results in improved adult spatial performance compared to controls without this exposure (Keeley et al., 2010; Wartman et al., 2012b) (3) exposure to environmental toxins from PND16 to PND20 reduces the *stratum oriens* projection in male Long Evans rats (Smith et al., 2011; Smith and Holahan, 2014) and (4) estradiol treatment in female rats from PND19 to PND22 improves spatial retention during adolescence (Wartman et al., 2012b).

## Functional Plasticity and Calcium

**FIGURE 4:**
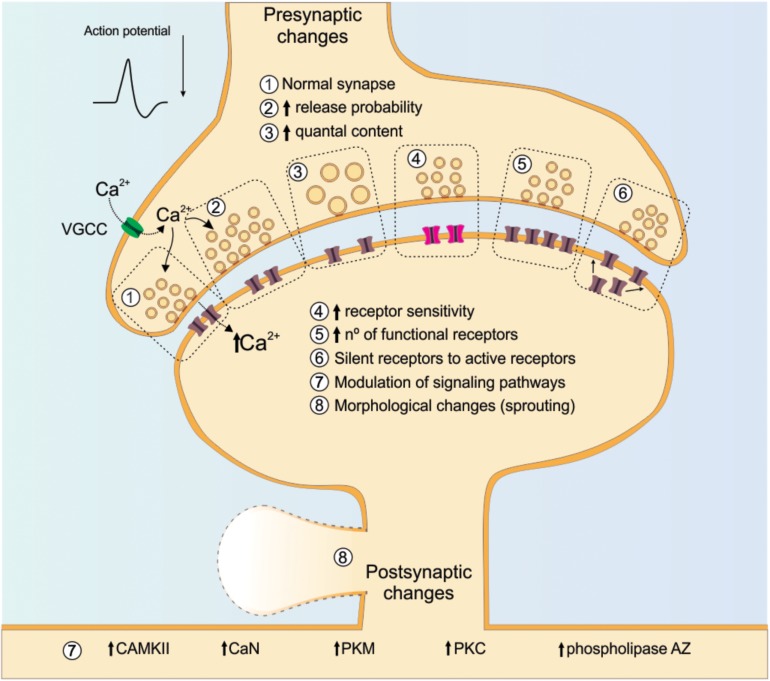
Cellular and molecular changes involved in synaptic plasticity. The figure illustrates numerous mechanisms that could be involved in synaptic plasticity. Special attention is given to Ca^2+^, as its increase can trigger either the LTP or LTD occurrence with evident intracellular signaling cascade. On the post-synaptic site, the illustrated receptor are NMDA and the silent receptors are AMPA. NMDA receptor: *N*-methyl-D-aspartate receptor; AMPA receptor: α-Amino-3-hydroxy-5-methyl-4-isoxazolepropionic acid receptor; VGCC: voltage-gated calcium channels; CaMKII: calcium/calmodulin-dependent protein kinase type II; CaN: calcineurin; PKC: protein kinase C, PKM: protein kinase M.

An important mediator of both pre- and post-synaptic functional plasticity, and thereby crucial for the long-term changes that ensue following successful completion of sensitive developmental periods is intracellular calcium (Ca^2+^). A depolarizing event produced by stimulation of the presynaptic component leads to an increase in the probability that VGCCs will open and allow the influx of Ca^2+^ (Nanou and Catterall, 2018). One role for Ca^2+^ influx through presynaptic VGCCs is the initiation of secretory processes for neurotransmitter release from presynaptic terminals (Ackermann et al., 2015). On the post-synaptic side, rises in Ca^2+^ concentrations trigger a cascade of intracellular processes involving activation of protein kinase C (PKC), protein kinase M (PKM), Ca^2+^/calmodulin-dependent kinase II (CaMKII), phospholipase A2 and other calcium-dependent processes (Herring and Nicoll, 2016; Lisman, 2017; Borovac et al., 2018) (Figure 4).

**FIGURE 5:**
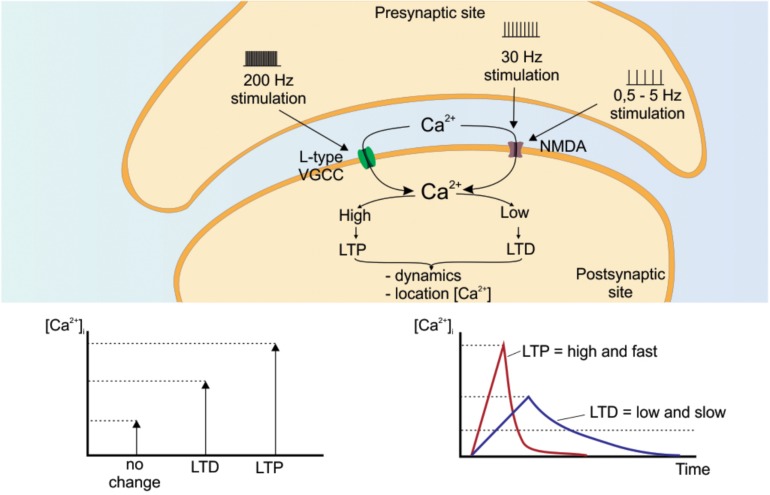
Intracellular calcium dynamics to induce LTP/LTD. Several factors related to Ca^2+^ dynamics influence long-term changes in synapses, strengthening or weakening the transmission of information in this structure. LTP or LTD are influenced by the amplitude, duration and location of calcium signaling. Variations in these factors allow different paradigms that may explain how the same signaling element may lead to different cellular and molecular responses. NMDA receptor: *N*-methyl-D-aspartate receptor; VGCC: voltage-gated calcium channels.

Intracellular Ca^2+^ dynamics are linked to the main cellular mechanisms that may underlie learning and memory and plasticity in general. Lasting changes in the strength/weakening of the synapses, often brought about by changes in intracellular Ca^2+^, are essential for memory storage (Izquierdo, 1993; Bortolotto et al., 2001; Izquierdo et al., 2019) and causally related to LTP and LTD. For example, spike timing-dependent plasticity – STDP (for review, Sjöström and Nelson, 2002), requires robust rises in intracellular Ca^2+^ and a combination of pre- and post-synaptic activation to trigger intracellular mechanisms leading to gene expression and protein synthesis (Figure 5) (Sjöström and Nelson, 2002; Malenka and Bear, 2004; Luscher and Malenka, 2012). In hippocampal CA1 pyramidal neurons, Ca^2+^ enters the post-synaptic site throughout different channels, such as NMDA and/or VGCC, to induce LTP (Evans and Blackwell, 2015). LTP can be induced by high frequency stimulation around 200 Hz, which would require VGCC. On the other hand, frequencies of about 30 Hz activate NMDA receptors to induce LTP (Sjöström and Nelson, 2002; Malenka and Bear, 2004; Luscher and Malenka, 2012). In this sense, Moosmang et al. (2005) showed decreased VGCC-dependent LTP without alteration on NMDA-dependent LTP in the CA1 hippocampal neurons using L-Type VGCC (Ca_v_1.2) knockout animals. Ca_v_1.3 knockout mice, another subtype of L-Type VGCC, did not change the 200Hz-induced LTP in CA1 hippocampal neurons (Clark et al., 2003) but did impair NMDA-dependent LTP using a 100 Hz stimulation in amygdala neurons (McKinney et al., 2009).

Contrary to what a happens in LTP, LTD may be induced by low-frequency synaptic stimulation (0.5–5 Hz) and a combination of pre- and post-synaptic activation. These factors together or alone will cause small intracellular Ca^2 +^ elevation that comes from glutamate receptors, leading to depression in the post-synaptic response (Sjöström and Nelson, 2002; Evans and Blackwell, 2015). Not only is Ca^2+^ influx amplitude important for LTP/LTD, but cytoplasmic dynamic and intracellular event location (for details see Sjöström and Nelson, 2002; Evans and Blackwell, 2015) (Figure 5). Therefore, the neural attributes (e.g., VGCCs or *N*-methyl-D-aspartate – NMDA receptors) that allow Ca^2+^ influx into pre- and post-synaptic compartments must be in place prior to the emergence of any sensitive developmental period for these changes to have sustained, long-lasting effects.

### Calcium Channels in the Hippocampus

The development and expression of VGCCs within hippocampal neurons has been reported to be phasic, and coincides with, or may even mediate, increased cognitive processes. Using the single-electrode voltage clamp technique, Krnjevic et al. (1989) assessed the development of voltage-dependent inward and outward currents of Ca^2+^ channels within the hippocampus. While they did observe inward Ca^2+^ currents in rats as young as PND1, the current in young rat pups (PND1-6) was 65–95% lower than adult rats. Depolarizing pulses did, however, evoke inward Ca^2+^ currents in PND7-13 rats that were not significantly different than those in adult neurons (Krnjevic et al., 1989). They further noted that there was no evidence of an initial predominance of T-type current (predominantly somatic), with an L-type current (predominantly dendritic) appearing later in development coinciding with dendritic maturation (Krnjevic et al., 1989; Erecinska et al., 2004).

Using radioligand binding of N-type Ca^2+^ channels, Jones et al. (1997) aimed to establish a developmental timeline of these Ca^2+^ channels from the hippocampus in rats. The earliest binding was detected in embryonic (E)18. From E18 to PND6, the binding increased at a constant rate of 0.025 pmol/mg per day. Increases in binding were more gradual after PND6, reaching a peak at PND16, then declining to levels seen in adults (Jones et al., 1997). To complement these findings, N-type Ca^2+^ channel expression within the hippocampus at similar developmental time-points using Fl-ω-CgTx labeling was carried out (Jones et al., 1997). Little to no labeling was observed at E19, but by PND0, labeling was observed in CA3 and CA4, the somata of the subiculum, and the external granule cell layer of the dentate gyrus. Labeling in CA1 and CA2 only began to appear at around PND4, along with the internal granule cell layer of the dentate gyrus, with expression reaching adult levels throughout all hippocampal subfields thereafter (Jones et al., 1997). They postulated that the discrepancy in labeling between CA1 and CA2 and adjacent regions may result from differential translation and insertion of N-type Ca^2+^ channels at the nerve surface of neurons. The lack of N-type Ca^2+^ channel expression, despite the presence of dendrites, suggests that these channels are also inserted into the dendritic membrane, and are therefore expressed on dendrites only after these regions are mature enough to support the mechanisms responsible for trafficking, insertion, and immobilization of these channels (Jones et al., 1997).

### Calcium Channels in Other Structures

Development of VGCCs in other brain regions has been shown to mirror that of the hippocampus. High-VGCCs within Cajal-Retzius, subplate, and pyramidal cells of the somatosensory cortex in PND0 – 5 rats were assessed using whole-cell patch-clamp recordings (Luhmann et al., 2000). They reported that high-voltage-gated Ca^2+^ currents were detected in cells within the somatosensory cortex in rats as young as PND0. The pyramidal neurons, which showed the highest Ca^2+^ current peak, largest peak current density, and highest steady-state current density, are migrating neurons, while non-migrating subplate and Cajal-Retzius cells showed the lowest peak current density (Luhmann et al., 2000). The differences in Ca^2+^ current peak amplitude may outline the contribution of Ca^2+^ channels to the early maturation of the cerebral cortex, given that cellular migration, for example, has been shown to be highly dependent on intracellular Ca^2+^ concentrations (Komuro and Rakic, 1992; Luhmann et al., 2000).

In cerebellar Purkinje cells, Liljelund et al. (2000) reported sustained spontaneous oscillations of intracellular Ca^2+^ in rats as early as PND4, a prominent feature of early developing Purkinje cells. They used specific inhibitors for P/Q-, N-, and L-type Ca^2+^ channels to determine whether these oscillations were dependent on Ca^2+^ influx through VGCCs (Liljelund et al., 2000). While there were no significant differences in oscillations in the P/Q- or N-type channels, the amplitude of Ca^2+^ oscillations through the L-type channels significantly decreased. This was unexpected as P-type channels are known to play a predominant role in Ca^2+^ signaling in dendrites of mature Purkinje cells and blockade of those channels have been shown to completely inhibit Ca^2+^ currents in both immature and mature Purkinje neurons (Hillman et al., 1991; Mintz et al., 1992; Usowicz et al., 1992; Liljelund et al., 2000). To confirm the expression of these subtypes at these early developmental stages, immunostaining was used and equally intense staining on the soma for all subtypes was reported (Liljelund et al., 2000). They concluded that, while all three Ca^2+^ channel subtypes were expressed on the soma of immature Purkinje neurons in PND4-7 rats, L-type channels, but not P- nor N-type, were the predominant channel involved in Ca^2+^ oscillation generation and L-type channel activation increased the expression of immediate early genes, which is important for signaling in developing Purkinje neurons (Liljelund et al., 2000).

A more variable degree of expression and function of Ca^2+^ channel subtypes within the cerebellum, thalamus, and neocortex throughout various stages of development has been reported (Iwasaki and Takahashi, 1998). Stimulating Purkinje cell axons and inhibiting N-type Ca^2+^ channels at PND7 caused a partial blockade of inhibitory post-synaptic current (IPSC) amplitude, while P/Q-type channel inhibition abolished the remaining fraction of IPSCs (Iwasaki and Takahashi, 1998). However, inhibiting N-type channels resulted in a progressively lessened effect on IPSCs, until it was eventually lost at PND16, whereas inhibiting P/Q-type channels abolished IPSCs in rats older than PND16 (Iwasaki and Takahashi, 1998). These results suggest that, while multiple Ca^2+^ channels are involved in synaptic transmission in Purkinje cells at PND6 – 8, a switch occurs during development, whereby inhibitory transmission from Purkinje cells to deep nuclear cells is exclusively mediated by P/Q-type channels. The same observations were reported in thalamocortical relay neurons in the laterodorsal thalamic nucleus, indicating that this switch may be common among many central synapses (Iwasaki and Takahashi, 1998). To establish whether the disappearance of these channels or a decoupling of presynaptic Ca^2+^ channels caused the reduced effect of N-type channel blockade, they recorded Ca^2+^ currents directly from the presynaptic terminal, the calyx of Held, in the brainstem. The N-type channels were still present at PND7 at the presynaptic terminal but were significantly reduced at PND10, while the expression of P/Q-type channels increased (Iwasaki and Takahashi, 1998). At PND13, the N-type channels were completely lost from the calyceal presynaptic terminals and replaced by P/Q channels. This reduction in the contribution of N-type channels at various central synapses was not found to be universal. No change in the relative contribution of N-type calcium channels to excitatory post-synaptic currents (EPSC) in geniculo-cortical synapses was observed between PND10 and PND40, suggesting that both N-type and P/Q-type channels mediate synaptic transmissions at this synapse throughout postnatal development (Iwasaki and Takahashi, 1998).

These reports demonstrate that VGCCs are present and functionally active well before the sensitive developmental period for the emergence of spatial function in rodents. This would allow for the requirement of elevated Ca^2+^ levels and downstream actions for the sustained maintenance of the plastic processes that occur during this sensitive developmental period.

## Glucose-Mediated Calcium Regulation

**FIGURE 6:**
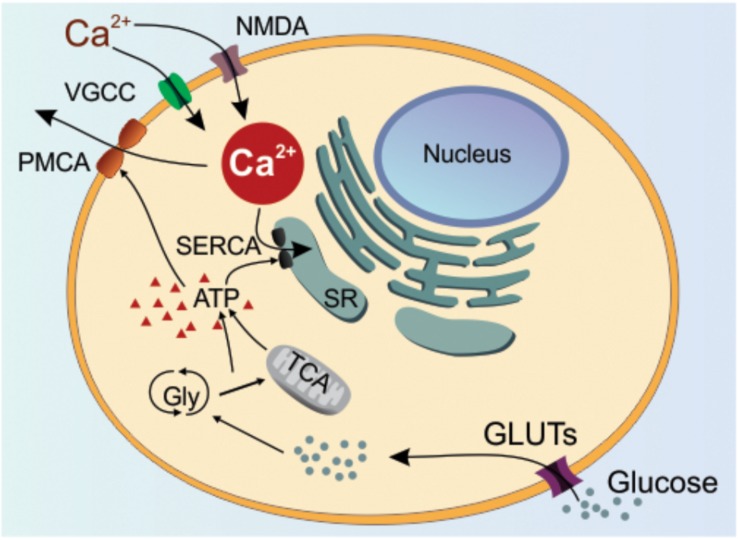
Glucose-regulation of Calcium. In the physiological situation, glucose is mainly transported by glucose transporters (GLUTs) located in the neuronal plasma membrane. The glucose is then converted to ATP by glycolysis and tricarboxylic acid cycle (TCA). The produced ATP will serve as a substrate for two ATPases, SERCA and PMCA, which pump Ca^2+^ into the endoplasmic reticulum and out of the cell, respectively. Ca^2+^ has its influx into the cytoplasm by various cellular mechanisms, however only VGCC (voltage-gated calcium channel) and NMDA (*N*-methyl-D-aspartate) receptors are represented by their importance to LTP/LTD. Changes in the glucose transport or metabolism by the neuron are expected to impact ATP-dependent mechanisms leading to intracellular Ca^2+^ deregulation. SR: sarcoplasmic reticulum; PMCA: plasma membrane Ca^2+^-ATPase; SERCA: sarco/endoplasmic reticulum Ca^2+^-ATPase; Gly: glycolysis; ATP: adenosine triphosphate.

Different levels of Ca^2+^ can influence whether a neuron undergoes LTP or LTD (Sjöström and Nelson, 2002; Evans and Blackwell, 2015). It is also well-established that supra-high levels of intracellular Ca^2+^ will set up conditions whereby the neuron becomes vulnerable to cell death processes (Núñez and Hidalgo, 2019). To control for supra-high Ca^2+^ levels, appropriate energy resources are required to regulate Ca^2+^ so that there are beneficial outcomes such as sustained plasticity. An important component of this regulatory mechanism is the transport of glucose (Figure 6) (Thayer et al., 2002; Ivannikov et al., 2010; James et al., 2013; Mergenthaler et al., 2013; Dienel, 2019).

### Glucose Changes During Development

One neural attribute whose function may be altered during development, in response to early life experience and provide a mechanism to preserve memory through the lifespan is glucose utilization. Glucose is a carbohydrate that fuels neural activity to permit functional and structural changes in neurons associated with plasticity and memory processes. Several reports have shown that glucose metabolism increases from birth to adulthood with a significant peak around PND14 to PND17 (Nehlig, 1997; Vannucci and Vannucci, 2000). Because these peak increases in glucose utilization overlap with the timeframe during which spatial training can enhance later memory processing (Keeley et al., 2010; Wartman et al., 2012a), it might be the case that learning-associated changes in glucose utilization would provide an important neural functional change to preserve memory function throughout the lifespan.

It has been shown that increased glucose ingestion during childhood can positively impact functional plasticity in humans as measured via enhanced learning and memory (Benton and Stevens, 2008). However, sustained hyperglycemia can have detrimental effects during development on later cognitive function (Ryan and Williams, 1993; Malone et al., 2006). Likewise, hypoglycemia during development can lead to abnormalities in neurocognitive function from mild dysfunction to severe mental retardation (Flykanaka-Gantenbein, 2004). The importance of a glucose-dependent regulated period during childhood becomes more evident when following Type 1 diabetes patients (insulin-dependent; juvenile diabetes) who experience hypo- or hyperglycemic states (Hershey et al., 2003, 2005; Perantie et al., 2008) and show marked decline in brain function and cognitive tasks later in life.

Previously, we have shown that impairment in glucose metabolism can have a detrimental impact on neurons and their intrinsic properties leading to cell death (Oliveira et al., 2007; Cruz et al., 2012; Moreira-Lobo et al., 2017). By reducing thiamine which works as a cofactor required for glucose metabolism, we impaired ion channel properties and functional cellular outcomes ultimately leading to neural cell death (Cruz et al., 2012). Curiously, thiamine deficiency in pregnant female rats leads to similar neurodegenerative changes in the neurons of offspring as those seen *in vitro* (Oliveira et al., 2007).

### Glucose Availability and Intracellular Calcium

Glucose is the main source of energy for neural function, so any reduction in glucose metabolism or availability will affect ATP-dependent cellular mechanisms. One of the most important ATP-dependent neuronal mechanism includes the intracellular Ca^2+^ buffering, which is tightly adjusted to maintain intracellular Ca^2+^ levels at rest and during cellular activity (Mergenthaler et al., 2013; Chen et al., 2015; Stincone et al., 2015; Szablewski, 2017; Dienel, 2019). Neurons use ATPases to pump the cytoplasmic increase of Ca^2+^ to the endoplasmic reticulum and out of the cell. This pumping occurs through two ATPases: the PMCA (Plasma Membrane Ca^2+^-ATPase) and SERCA (Sarco/Endoplasmic Ca^2 +^-ATPase) (Brini and Carafoli, 2009; Schnellmann and Covington, 2010; Calì et al., 2017; Chemaly et al., 2017). For this reason, in the absence of an adequate supply of ATP, which is mainly provided by glycolysis and mitochondria, Ca^2+^ levels may increase leading to deleterious cellular effects or loss of function (Figure 6) (Berridge, 1998; Blass, 2000; Calì et al., 2017, 2018).

Serum Ca^2+^ concentrations are highest in neonates and infants, decreasing over childhood and adolescence, and become stable at the age of 17 years (Naganathan and Gossman, 2017). However, it’s not clear how Ca^2+^ is handled by the cell during the childhood period (Liu et al., 2014; Dai et al., 2015; Alviña et al., 2016), essentially due to drastic changes in glucose over this period of time (Nehlig, 1997; Vannucci and Vannucci, 2000).

### Glucose-Regulation of Calcium and the Lifespan

Progress in understanding the changes in neural glucose metabolism during the pathological processes of AD has grown rapidly in recent decades leading to the conclusion that there is hypometabolism in certain brain regions following cognitive decline associated with AD pathology (Costantini et al., 2008; Cunnane et al., 2011). Because glucose hypometabolism is a contributing factor during the asymptomatic stage and the initial development of AD, enhancing glucose utilization during critical stages of development (e.g., preadolescence) may serve to protect against cognitive decline as observed in AD.

Dysfunction of glucose metabolism may be a confluent point in several brain diseases (Pratt et al., 2008; Nijland et al., 2014, 2015; Dean et al., 2016; Anandhan et al., 2017; Lauretti et al., 2017; Szablewski, 2017). One of the most important dementias related to glucose hypometabolism is AD (Costantini et al., 2008; Cunnane et al., 2011; Jack et al., 2013, 2014), which interestedly also presents disturbances in the regulation of the dynamics and signaling of cellular Ca^2+^ (Disterhoft et al., 1994; Mattson and Chan, 2003; Bezprozvanny and Mattson, 2008; Wykes et al., 2012; Gibson et al., 2017). However is unclear if reduced glucose metabolism and Ca^2+^ deregulation have any causal relationship in AD, though both conditions can directly impact cognition (McKay et al., 2009; Owen and Sunram-Lea, 2011; Oh et al., 2013; Berridge, 2014; Morris et al., 2014). It will be possible that early experience establishes not only multiple neuronal pathways within the hippocampus, which will help protect against memory decline later in life (Wartman et al., 2012a) but also changes in glucose utilization that will aid in optimal Ca^2+^ regulation during the aging/neurodegenerative process. From other perspectives, several papers show that stress or disturbing during childhood can directly impact the following life (Dunn et al., 2013; Watt et al., 2017; Andersen, 2019), showing that the period of childhood might subside important changes in adulthood or later.

## Future Directions and Conclusion

One important question in the context of preserving functional plasticity over the lifespan is the determination of the neurobiological functional changes that occur during sensitive developmental periods that might protect against later memory dysfunction by promoting the establishment of redundant neural pathways. The main question being, if plasticity during the childhood period does strengthen and preserve memory stability over the lifespan, what are the neurobiological structural or functional substrates that mediate this effect? In previous work from our lab (Wartman et al., 2012b), separate groups of preadolescent (18–26 days old) Long Evans rats were given spatial training on either a water-maze or a dry-maze or received no exposure to the spatial cues. Three weeks later, rats were tested on the water maze or dry maze tasks. When animals experienced any spatial training during the preadolescent period, there was improved performance during the adolescent period compared to animals with no preadolescent spatial training. Compared to groups with no preadolescent spatial exposure, groups with preadolescent spatial training showed elevated neural connectivity patterns in the hippocampus during adolescence indicating an expanded neural network in this region. It was hypothesized that preadolescent training may confer a more complex neural network that facilitates information processing thereby leading to enhanced memory function during the adolescent period. The initial and end time point of the beneficial learning window couple perfectly within the beginning and the end of the childhood period described and discussed above. Based on these results it is clear that childhood is a labile time period where any influence could modulate cellular and molecular signaling shaping later behavioral outputs. Together these data show that during the childhood period, we may have opportunities to control cellular and molecular pathways to enhance cognition throughout the lifespan.

While these studies only examined the adolescent time point, future studies should address the stability of these neural circuit reorganizations that may serve to facilitate information processing over the lifespan, in particular, during the aging process or neurodegenerative processes. In addition, these studies mainly focused on structural changes so one remaining question is whether there are concurrent functional changes in glucose metabolism or Ca^2+^ homeostasis that would serve to preserve memory function over the lifespan.

One neural function that might be altered with early life experience and provide a mechanism to preserve memory through the neurodegenerative process is glucose utilization. Several reports have shown that glucose metabolism increases from birth to adulthood with a significant peak around 14–17 days old (Nehlig, 1997; Vannucci and Vannucci, 2000). Because these peak increases in glucose utilization overlap with the timeframe during which spatial training can enhance later memory processing, it might be the case that learning-associated changes in glucose utilization would provide an important neural functional change to preserve memory function throughout the lifespan. In this sense, data about glucose metabolism during the pathological processes of AD have grown rapidly in recent decades leading to the conclusion that there is hypometabolism in certain brain regions following cognitive decline associated with AD pathology (Costantini et al., 2008; Cunnane et al., 2011). Because glucose hypometabolism is a contributing factor during the asymptomatic stage and the initial development of AD, enhancing glucose utilization during critical stages of development (e.g., preadolescence) may serve to protect against cognitive decline as observed in AD.

Based on assumption that glucose hypometabolism and Ca^2+^ clearance in the cell have a functional link, it is not clear as to whether dysregulated Ca^2+^ signaling via changes in glucose utilization are the primary cause or secondary effect of memory loss associated with AD, we hypothesize that enhancing glucose utilization with spatial training during the preadolescent period would provide a functional enhancement that regulates glucose-dependent Ca^2+^ regulation during aging or neurodegeneration and provide essential neural resources to preserve memory function. Specifically, we hypothesize that early experience establishes not only multiple neuronal pathways within the hippocampus, which will help protect against memory decline later in life, but also changes in glucose utilization that will aid in optimal Ca^2+^ regulation during the aging/neurodegenerative process.

We also predict that early cognitive training can mitigate the memory-associated deficits associated with AD neuropathology. Training animals on a spatial task during the preadolescence period would relieve/retard the memory associated deficits of AD pathology later in life. Besides that, we hypothesize that AD animals would show memory deficits and associated reductions in levels of GLUT3, GLUT1 and Ca_v_1.2 while animals given early training would show persevered levels of GLUT3, GLUT1 and Ca_v_1.2 compared to animals that are not given early training and are not under the memory loss spectrum.

The combination of neuroanatomical and behavioral results from the reviewed work leads to the hypothesis that PND18–PND21 represents a sensitive period for hippocampal development and modification and the emergence of allocentric/spatial cognitive function. Glucose-mediated regulation of Ca^2+^ concentrations inside the cell appears to be a critical factor in stabilizing this time of high plasticity. One might speculate that with high rates of synaptic modification occurring during this developmental period, memory formation, thought to depend on synaptogenesis, would proceed efficiently. Yet, because of the ever-changing neuronal landscape due to pruning, some structural modifications hypothesized to underlie memory formation (e.g., axon and spine synaptogenesis) might be lost with ensuing memory deficits. A question for future research would be to determine the relative memory storage versus loss that might occur during this period of postnatal hippocampal modification.

This review has aimed to uncover how, amidst constant metabolic turnover in the brain, memories are stored and become stable, durable and persistent through time. The novelty and significance of this information are that little is known concerning the mechanisms underlying memories processed and stored by the juvenile, developing the mammalian brain. Experiments on this topic will contribute to the field of memory by identifying the crucial structures, proteins and metabolic changes that maintain functional plasticity throughout the lifespan. This approach is innovative in that it represents a new idea in the study of early life memory processing during development related to early changes in synaptic organization and glucose metabolism. Characterizing these changes via a longitudinal study across many days will contribute to understanding shifts in memory storage. Combined, these contributions will help provide the basic knowledge required to address issues of memory enhancements and deficits.

## Author Contributions

FO and MH initiated a collaboration to explore this topic of development and plasticity through the lifespan and contribution of glucose-mediated Ca^2+^ regulation. FO wrote the sections on aging and glucose. MH wrote the developmental sections and concluding remarks. NT wrote the section on VGCCs. All authors edited and revised the manuscript and approved the final version.

## Conflict of Interest Statement

The authors declare that the research was conducted in the absence of any commercial or financial relationships that could be construed as a potential conflict of interest.
